# IgA nephropathy relapse following COVID-19 vaccination treated with corticosteroid therapy: case report

**DOI:** 10.1186/s12882-022-02769-9

**Published:** 2022-04-07

**Authors:** Shota Watanabe, Shuling Zheng, Arash Rashidi

**Affiliations:** 1 Internal Medicine, University Hospitals Cleveland Medical Center, Case Western Reserve University, 11100 Euclid Ave, Cleveland, OH 44106 USA; 2 Pathology, University Hospitals Cleveland Medical Center, Cleveland, USA; 3 Nephrology, University Hospitals Cleveland Medical Center, Cleveland, USA

**Keywords:** COVID-19 vaccine, IgA nephropathy, AKI, Hematuria, Kidney biopsy

## Abstract

**Background:**

The flare of immune-mediated disease following coronavirus disease of 2019 (COVID-19) vaccination is a rare adverse event following immunization. De novo, as well as relapsing IgA nephropathy (IgAN) cases, have been reported following either mRNA-1273 (Moderna) or BNT162b2 (Pfizer-BioNTech) vaccination. To our knowledge, the majority of IgAN relapses did not result in severe acute kidney injury (AKI) and resolved spontaneously.

**Case presentation:**

This is a case of a 54-year-old female with a previous diagnosis of IgAN who developed IgAN relapse following the second dose of Moderna vaccine. Gross hematuria developed 2 days after vaccination, which was accompanied by significant AKI. Kidney biopsy showed mild tubular atrophy and IgA staining in mesangium without crescent formation. Significant improvement in serum creatinine (Cr) was observed on day 10 after initiating prednisone. Cr came back to normal within 3 months after initiating corticosteroid.

**Conclusion:**

COVID-19 vaccination is associated with a flare of IgAN that may cause significant AKI. Steroid therapy is associated with recovery. IgAN flare after COVID-19 vaccination should be closely monitored to elucidate any adverse effect associated with the novel vaccine.

**Supplementary Information:**

The online version contains supplementary material available at 10.1186/s12882-022-02769-9.

## Background

The flare of immune-mediated disease (IMD) following coronavirus disease of 2019 (COVID-19) vaccination is a rare adverse event following immunization. Previous studies reported flare-up of various IMDs, such as rheumatoid arthritis, systemic lupus erythematosus (SLE), Behcet’s disease, psoriasis, vasculitis, sarcoidosis, and multiple sclerosis [1–4]. De novo, as well as relapsing IgA nephropathy (IgAN) cases, have been reported following either mRNA-1273 (Moderna) or BNT162b2 (Pfizer-BioNTech) vaccination [5–10]. The majority of relapsed IgAN in previous reports did not result in severe acute kidney injury (AKI) and resolved without intervention (Table 1). However, Plasse et al. reported an IgAN relapse following the second dose of Pfizer vaccine, which caused significant AKI and subnephrotic range proteinuria. Kidney biopsy was not reported. AKI resolved 1 month after starting steroid therapy, and proteinuria returned to baseline level within 2 months [11]. Here we report an IgAN relapse with significant AKI after administration of Moderna vaccine, which resolved after initiating steroid therapy. Renal biopsy was performed to rule out other de-novo glomerulonephropathies.

**Table 1:** IgAN relapses following COVID-19 vaccination

Author	Age (Years)	Sex	Manufacture	Dose (1^st^/2^nd^)	Time between vaccine and onset (days)	Presentation	Pathology	Treatment	Response
**Gul Rahim**	52	F	Pfizer	2nd	< 1	GH, AKI	N/A	None	Remission of GH in less than 1 week
**Negrea**	38	F	Pfizer	2^nd^	< 1	GH, SRP	N/A	None	Remission of GH after 3 days
	38	F	Pfizer	2^nd^	< 1	GH	N/A	None	Remission of GH after 3 days
**Perrin**	22	M	Moderna	1^st^	2	GH	N/A	None	Remission of GH
	41	F	Pfizer	1^st^	2	GH	N/A	None	Remission of GH
	27	F	Pfizer	2^nd^	2	GH	N/A	None	Remission of GH
**Plasse**	N/A	N/A	Pfizer	2^nd^	5 to 6	GH, SRP, AKI	N/A	Corticosteroids	Remission of GH, AKI after 1 month, SRP within 2 months
	N/A	N/A	Pfizer	2^nd^	1	GH	N/A	None	Remission of GH after 3 days
**This Case**	54	F	Moderna	2^nd^	2	GH, SRP, AKI	Active IgAN	Corticosteroids	Remission of GH after 2 days, AKI in 3 months

*Abbreviations: AKI* Acute Kidney Injury, *GH* Gross Hematuria, *SRP* Subnephrotic Range Proteinuria

## Case presentation

A 54-year-old, Caucasian female with history of IgAN after strep throat infection that was diagnosed with renal biopsy in 2006. Other significant co-morbidity includes obesity (BMI 31.6), hypertension, and GERD. She had no prior documented infection with COVID-19. She was on enalapril 20 mg daily, hydrochlorothiazide 12.5 mg daily, and propranolol 120 mg daily. Her baseline creatinine level (Cr) was 1.2 (eGFR 46 mL/min/1.73m^2^). Urinalysis was positive for 2 + protein, 3 + blood, and red blood cell (RBC) 15 /high-power field (HPF). The total urine protein to Cr ratio was 1.03.

**Fig. 1:**
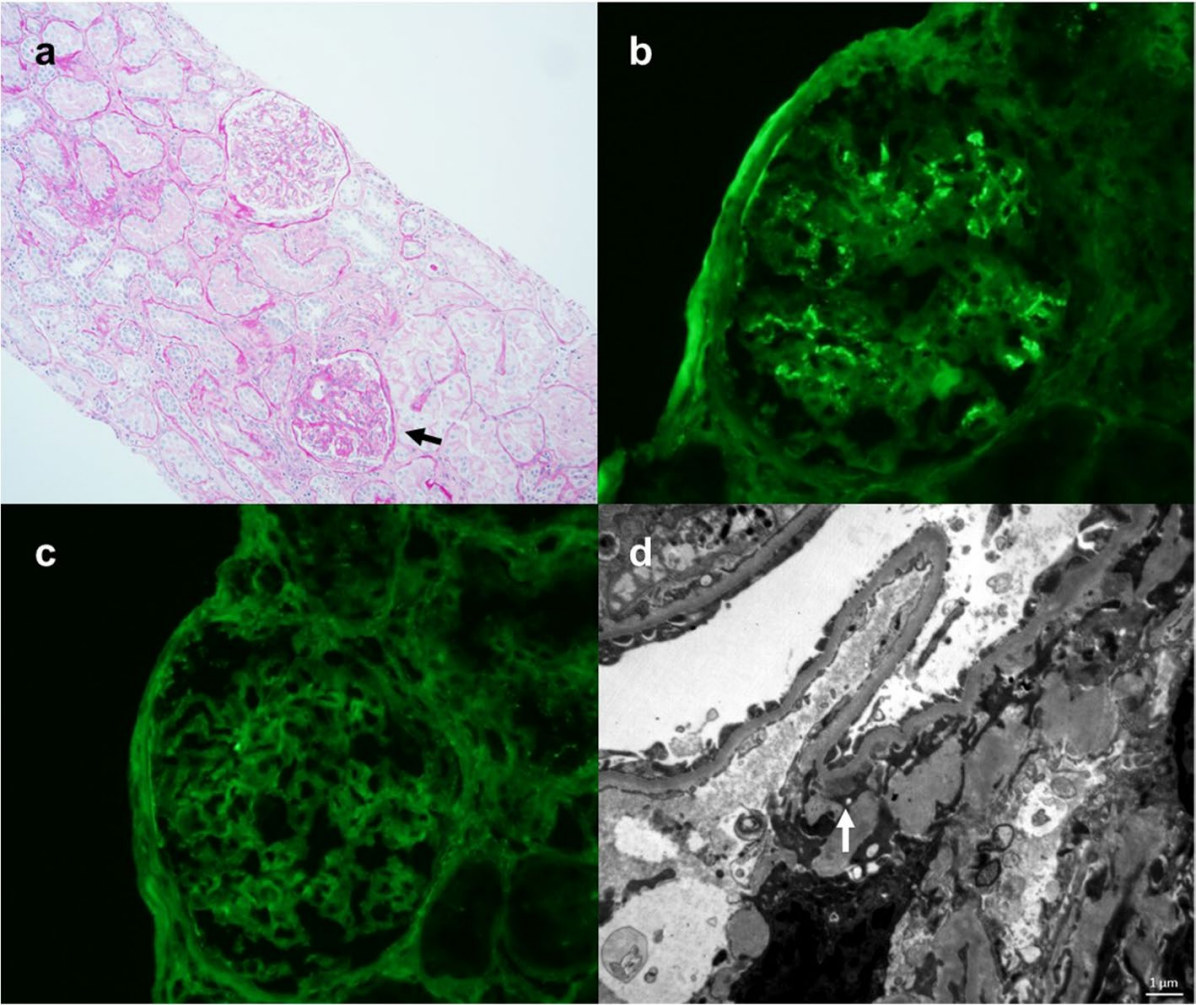
Histopathologic findings from renal biopsy. **a** Light microscopy shows no mesangial or endocapillary hypercellularity, or crescents. Fibrous adhesion to the Bowman capsule is identified focally (black arrow). There is mild interstitial fibrosis and tubular atrophy (original magnification × 10). **b** Immunofluorescence analysis demonstrates weak IgA staining in mesangium (original magnification × 20). **c** Immunofluorescence analysis demonstrates negative IgG staining in mesangium (original magnification × 20). **d** Electron microscopy reveals a small number of mesangial electron-dense deposits, especially underneath paramesangial basement membranes (white arrow). Bar = 1 μm

Two days after receiving the second Moderna vaccine, she developed gross hematuria that resolved spontaneously after 2 days. Vital sign upon examination: body temperature 36.5 °C, blood pressure 122/88 mmHg, heart rate 78 beats/minute. Physical exam was unremarkable without lower extremity edema. Follow-up Cr increased to 3.04 (eGFR 16 mL/min/1.73m^2^) approximately one week after vaccination. The urinalysis showed 1 + protein, 3 + blood, RBC 50/ HPF. The total urine protein to Cr ratio was 0.67. The renal ultrasound was unremarkable. Repeat kidney biopsy showed mild interstitial fibrosis and tubular atrophy without crescent formation (Fig. 1a). Immunofluorescence analysis showed weak IgA staining in mesangium (Fig. 1b). IgG staining was negative (Fig. 1c). Electron microscopy revealed some mesangial electron-dense deposits (Fig. 1d). Differential diagnosis included IgAN relapse, other de-novo glomerulonephropathies, urinary tract hemorrhage with obstruction, and urinary tract infection, among other causes of hematuria and AKI; however, given her history and kidney biopsy result, IgAN relapse was thought to be the most likely cause.

She was started on prednisone 60 mg daily. Cr level improved to 1.9 after 10 days, at which point prednisone was decreased to 40 mg daily. Thereafter prednisone was tapered down gradually over 2 months. Serum Cr recovered to 1.07 approximately 3 months after starting the steroid therapy. Patient tolerated the treatment without significant adverse effect.

## Discussion

Here we report a case of IgAN relapse with significant AKI following COVID-19 vaccination that resolved after initiating steroid therapy. In accord with previously reported cases, gross hematuria occurred within a week after vaccination and resolved without intervention. The natural course of AKI due to IgAN following COVID-19 vaccination is unknown, but this case took a longer period for AKI to resolve compared to the case reported by Plasse et al. [11]. The efficacy of steroid therapy remains inconclusive; nevertheless, AKI seems to be reversible as in the cases thar are not related to COVID-19 vaccination. The previous retrospective study conducted by Kveder et al. in 2009, involving 584 adult patients, showed that all cases of AKI associated with IgAN and macroscopic hematuria resolved at a median follow-up of 15 months regardless of treatment status [12].

The pathogenesis of IgAN flare-up after COVID-19 vaccination is yet to be elucidated. The RNA vaccine has been shown to elicit antigen-specific, CD4^+^ and CD8^+^ T-cell responses producing multiple cytokines, including Interferon-ɣ, Tumor necrosis factor-α, and Interleukin-2 in animal studies [13]. A previous study showed early serum IgA rise after COVID-19 vaccination [14]. Hyperresponsiveness of IgA1 antibody was documented among those who developed IgAN flare following flu vaccine [15]. Similarly, COVID-19 vaccination may induce IgAN flare via IgA1 hyperresponsiveness to systemic cytokine.

## Conclusion

IgAN relapse with significant AKI is associated with COVID-19 vaccination, and systemic steroid therapy is associated with recovery. IgAN exacerbation after COVID-19 vaccination should be closely monitored to elucidate any adverse effect related to the novel vaccine.

## Supplementary Information

**Additional file 1.**

## Data Availability

Serum chemistry and urine study data used in this case report are available in the supplementary material.

## Abbreviations

AKI: Acute kidney injury
COVID-19: Coronavirus disease of 2019
Cr: Creatinine
eGFR: Estimated glomerular filtration rate
HPF: High-power field
IgAN: IgA nephropathy
IMD: Immune-mediated disease
RBC: Red blood cell
SLE: Systemic lupus erythematous
