# Supplementary material for: IgA nephropathy relapse following COVID-19 vaccination treated with corticosteroid therapy: case report
Source: BMC Nephrol. 2022 Apr 7;23:135. doi: 10.1186/s12882-022-02769-9 (PMC8988530; doi:10.1186/s12882-022-02769-9)
Supplement: Supplementary file 1 — Additional file 1. [file 12882_2022_2769_MOESM1_ESM.docx]

Table S1. Serum Chemistries

|  | **3/10/2022** | **4/23/2021** | **5/3/2021** | **5/7/2021** | **6/8/2021** | **8/17/2021** |
| --- | --- | --- | --- | --- | --- | --- |
| Serum Glucose,  mg/dL | 91 | 92 | 109 | 95 | 112 | 77 |
| Serum Na,  mmol/L | 141 | 133 | 137 | 140 | 138 | 142 |
| Serum K,  mmol/L | 3.4 | 4.2 | 4.0 | 3.6 | 3.5 | 3.2 |
| Serum Cl,  mmol/L | 103 | 96 | 104 | 106 | 102 | 105 |
| Serum Bicarbonate,  mmol/L | 28 | 25 | 23 | 24 | 26 | 28 |
| Serum BUN,  Mg/dL | 17 | 41 | 63 | 44 | 28 | 8 |
| Serum Cr,  mg/dL | 1.23 | 3.04 | 2.48 | 1.94 | 1.44 | 1.07 |
| eGFR, mL/min/1.73m^2^ | 46 | 16 | 20 | 27 | 38 | 53 |

Abbreviations: Na–Sodium, K–Potassium, Cl–Chloride, BUN–Blood urea nitrogen, Cr–Creatinine, eGFR–Estimated Glomerular Filtration Rate

Table S2. Urinalysis and Urine Chemistries

|  | **3/10/2022** | **4/27/2021** | **5/19/2021** | **6/17/2021** | **8/17/2021** |
| --- | --- | --- | --- | --- | --- |
| **Urinalysis** |  |  |  |  |  |
| Color | Yellow | Yellow | Yellow | Straw | Yellow |
| Specific Gravity | 1.012 | 1.009 | 1.008 | 1.003 | 1.010 |
| pH | 5.0 | 5.0 | 5.0 | 5.0 | 5.0 |
| Protein | 2+ | 1+ | Negative | Negative | Negative |
| Glucose | Negative | Negative | Negative | Negative | Negative |
| Blood | 3+ | 3+ | 3+ | 3+ | 3+ |
| Nitrite | Negative | Negative | Negative | Negative | Negative |
| Leukocyte Esterase | Negative | Negative | Negative | Negative | Negative |
| White Cells/ HPF | 5 | 4 | 2 | < 1 | 3 |
| Red Blood Cells/ HPF | 15 | 50 | 61 | 1 | 39 |
| **Urine Chemistries** |  |  |  |  |  |
| Total Protein (Spot),  mg/dL | 92 | 48 | 22 | 5 | 16 |
| Total Creatinine (Spot),  mg/dL | 89.6 | 71.7 | 63.9 | 20.4 | 104.0 |
| Total protein/ Creatinine Ratio (Spot) | 1.03 | 0.67 | 0.34 | 0.25 | 0.15 |

Abbreviations: HPF–High Power Field

Table S3. Timeline of Events

| Date | Event |
| --- | --- |
| 3/18/2021 | 1^st^ dose of Moderna Vaccine |
| 4/15/2021 | 2^nd^ dose of Moderna Vaccine |
| 4/27/2021 | Initiation of Steroid therapy |
| 5/3/2021 | Renal Biopsy |
